# ZAP-X Stereotactic Radiosurgery for Arteriovenous Malformations: Initial Experience of Three Patients

**DOI:** 10.7759/cureus.86082

**Published:** 2025-06-15

**Authors:** Patrick Pema, Triston Messer, Harshal Shah, Zakery Dabbagh, Michael Chaga, Wenzheng Feng, Tingyu Wang, Darra Conti, Jing Feng, Joseph Hanley, Shabbar Danish, Nitesh V Patel, Timothy Chen

**Affiliations:** 1 Neurosurgery, Hackensack Meridian Health, Hackensack, USA; 2 Neurological Surgery, Hackensack University Medical Center, Nutley, USA; 3 Neurosurgery, Jersey Shore University Medical Center, Neptune, USA; 4 Neurosurgery, Hackensack University Medical Center, Hackensack, USA; 5 Radiation Oncology, Jersey Shore University Medical Center, Neptune, USA; 6 Neurological Surgery, Jersey Shore University Medical Center, Neptune, USA

**Keywords:** arteriovenous malformation, avm, srs, stereotactic radiosurgery, zap-x

## Abstract

Arteriovenous malformations (AVMs) are complex cerebrovascular anomalies that carry a significant risk of hemorrhage, particularly in younger patients. Stereotactic radiosurgery (SRS) is a non-invasive treatment option, often employed for AVMs that are deep-seated or surgically inaccessible. The ZAP-X system (ZAP Surgical Systems, Inc., San Carlos, CA), a novel gyroscopic, self-shielded SRS platform, offers advanced beam geometry and real-time imaging, potentially enhancing treatment accuracy while minimizing radiation exposure to surrounding tissues.

This case series presents three patients with AVMs treated with ZAP-X SRS between March 2024 and September 2024 at a single institution. Target volume ranged from 4.84 to 11.56 cm^3^. Prescription dose ranged from 27 to 37.5 Gy in three or five fractions. Dosimetric parameters, treatment quality metrics, and early clinical and radiographic outcomes were assessed. All three patients tolerated treatment without significant acute side effects. Early follow-up imaging (4-12 months) demonstrated either reduction or stability in AVM volume, with resolution of presenting symptoms in two patients. Plan quality metrics met established standards, with conformity and gradient indexes ranging from 1.176 to 1.257 and 2.68 to 3.139, respectively. No radiation-induced complications were observed.

This initial clinical experience supports the feasibility, safety, and precision of ZAP-X SRS for treating brain AVMs. The system’s technical advancements, including multiangle gyroscopic beam delivery and real-time imaging, enabled precise dose delivery even in complex or high-grade lesions. These findings highlight the potential of ZAP-X as a frontline or adjunctive therapy for AVMs. Further prospective studies are needed to assess long-term obliteration rates and functional outcomes.

## Introduction

Stereotactic radiosurgery (SRS) may treat a variety of benign and malignant conditions. Since its advent, SRS has been a treatment option for arteriovenous malformation (AVM) [1,2]. An AVM is a tangle of abnormally connected arterial and venous vessels, creating a high-flow, low-resistance shunt prone to rupture [3]. In children and young adults, brain AVM rupture is a common cause of seizures, neurological deficits, and intracerebral hemorrhage, which can lead to death [3,4]. Current treatments for unruptured brain AVMs include observation with follow-up, embolization, surgical resection, and SRS, either alone or in combination [4]. Indications for different brain AVM interventions depend on the risks of rupture and hemorrhage compared with the risks of treatment. Multiple grading systems have been developed to evaluate these risks. The most commonly used system, the Spetzler-Martin grading scale, assesses risks associated with surgical intervention based on AVM size, location relative to eloquent brain areas, and venous drainage pattern [4,5].

For small (<3 cm in diameter), deep brain AVMs that are not suitable for surgical resection, SRS has been shown to be successful alone or in combination with other interventions [6]. SRS is non-invasive, can be performed in the outpatient setting, and has a low risk of acute complications, but it is held back by a lack of immediate improvement and by potential radiation injury [3]. Notably, SRS has shown success in the obliteration of Spetzler-Martin intermediate- and high-grade AVMs, but with a significant risk of neurological decline [5]. More research is needed on SRS for management of AVM-induced symptoms, including seizures, headaches, and visual field defects [7-9].

The ZAP-X gyroscopic SRS system (ZAP Surgical Systems, Inc., San Carlos, CA) is the latest innovation in SRS technology. This therapy option offers a number of benefits over traditional SRS. Its self-shielding apparatus allows for the installation of this device without the necessity for a vault, reducing cost and unnecessary radiation exposure to personnel. This machine also includes a gyroscopic design, increasing the specificity of the delivery of radiation through the use of multiple non-coplanar angles. Additionally, the ZAP-X platform offers real-time imaging guidance and automatic realignment. These improvements make this system an exciting and novel option for patients who need SRS. 

Due to its novel nature, our project and others that focus on the ZAP-X SRS system are extremely relevant to the management of AVMs, refinement of the ZAP-X technology, and to the field of SRS. Our report meaningfully contributes to the usage specificity of SRS and ZAP-X technology for AVMs and can allow physicians to treat this pathology and its symptoms with greater accuracy. Here, we present our initial experience with three patients who were treated using ZAP-X SRS for AVM. We aim to inform and expand the literature surrounding this novel method of SRS and its utilization for AVMs.

## Case presentation

Three patients with AVMs were treated using ZAP-X SRS at Jersey Shore University Medical Center (JSUMC) from March 2024 to September 2024. There were two female patients and one male patient. Written informed consent was obtained from all three participants. Data extraction included patient demographics and symptoms, tumor characteristics and diagnosis, radiation dosing and parameters, and postoperative outcomes. The study protocol was approved by the Hackensack Meridian Health Institutional Review Board, Pro2024-0029.

Patient characteristics

Patient 1 was a 35-year-old male who initially presented with an AVM that was discovered incidentally. Subsequently, he reported experiencing headaches and visual changes. His medical history included comorbidities such as a prior motor vehicle injury, hypertriglyceridemia, vitamin D deficiency, and cervicalgia. He had not undergone any previous treatments for his AVM. The AVM was located posterolaterally in the left temporal lobe. Imaging findings revealed a prominent vessel in the left occipital region, suggesting an additional feeding vessel from the posterior circulation. There was no evidence of acute intracranial hemorrhage, acute territorial infarction, mass effect, or midline shift. The AVM was measured at 4.4x3.5 cm and was classified as a Spetzler-Martin grade 3 with superficial venous drainage. He was provided with treatment options and ultimately selected radiotherapy. Physicians from the neurosurgery and radiation oncology departments confirmed this patient for ZAP-X designated SRS treatment.

Patient 2 was a 35-year-old female who presented with an incidental finding of AVM following symptoms of visual loss in both eyes. She described an episode characterized by darkness and shadows along the outer aspect of her left eye and the inner aspect of her right eye, determined to be right homonymous hemianopsia. Her medical history included these comorbidities: ocular migraine, fibroids, and pre-eclampsia. She had not undergone any prior treatments for her AVM. The AVM was located in the left posterior temporal lobe without any evidence of perinidal or intranidal aneurysms. Imaging findings showed no acute hemorrhage, mass effect, or midline shift. The AVM was measured at 3.5x1.9 cm and was classified as a Spetzler-Martin grade 3 AVM. Given her age, the size and location of the AVM, and its features, the neurosurgery and radiation oncology teams agreed upon a recommendation for ZAP-X SRS treatment for maximal disease control.

Patient 3 was a 27-year-old female who presented with an AVM and a history of migraines. Her medical history includes gastroesophageal reflux disease, arthritis, and anxiety/depression. She had undergone several embolization procedures for her AVM. These procedures were reportedly performed without complication. The AVM was located in the right parieto-occipital region and involved the midline and parasagittal frontoparietal areas and was larger on the right side than on the left. Imaging showed a partially embolized AVM with regional central and cortical venous drainage dilation. The AVM was measured at 6.0x3.2 cm and was classified as Spetzler-Martin grade 4. While her presentation was asymptomatic, this patient had undergone multiple embolizations due to the very large and complex nature of the AVM. Before SRS therapy, the patient was recommended to undergo an additional stage of embolization to decrease the flow to the AVM and make it more amenable to definitive treatment with SRS. The radiation oncology department then recommended ZAP-X-designated SRS treatment to the AVM nidus for disease control.

Imaging

**Figure 1:**
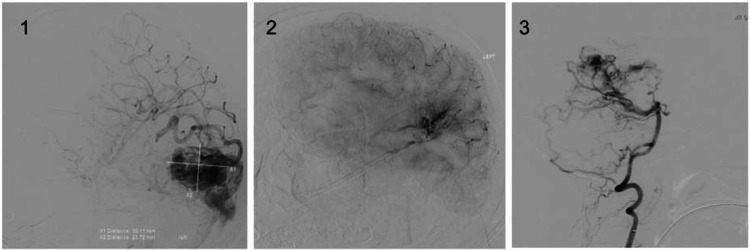
Preoperative cerebral angiography displaying the AVMs of patients 1 (1), 2 (2), and 3 (3). Image Credit: Patrick Pema AVM, arteriovenous malformation

Before treatment, the diagnosis of AVM was confirmed by the neurosurgery department through cerebral angiography. These images are displayed in Figure 1.

All three patients underwent preoperative MRI and CT scans for SRS treatment planning. MRI consisted of gadolinium contrast on a 0.5 T synaptive MRI using T1 3D spoiled gradient recalled, steady-state free precession, T2, and time-of-flight magnetic resonance angiography (MRA).

Treatment planning

**Figure 2:**
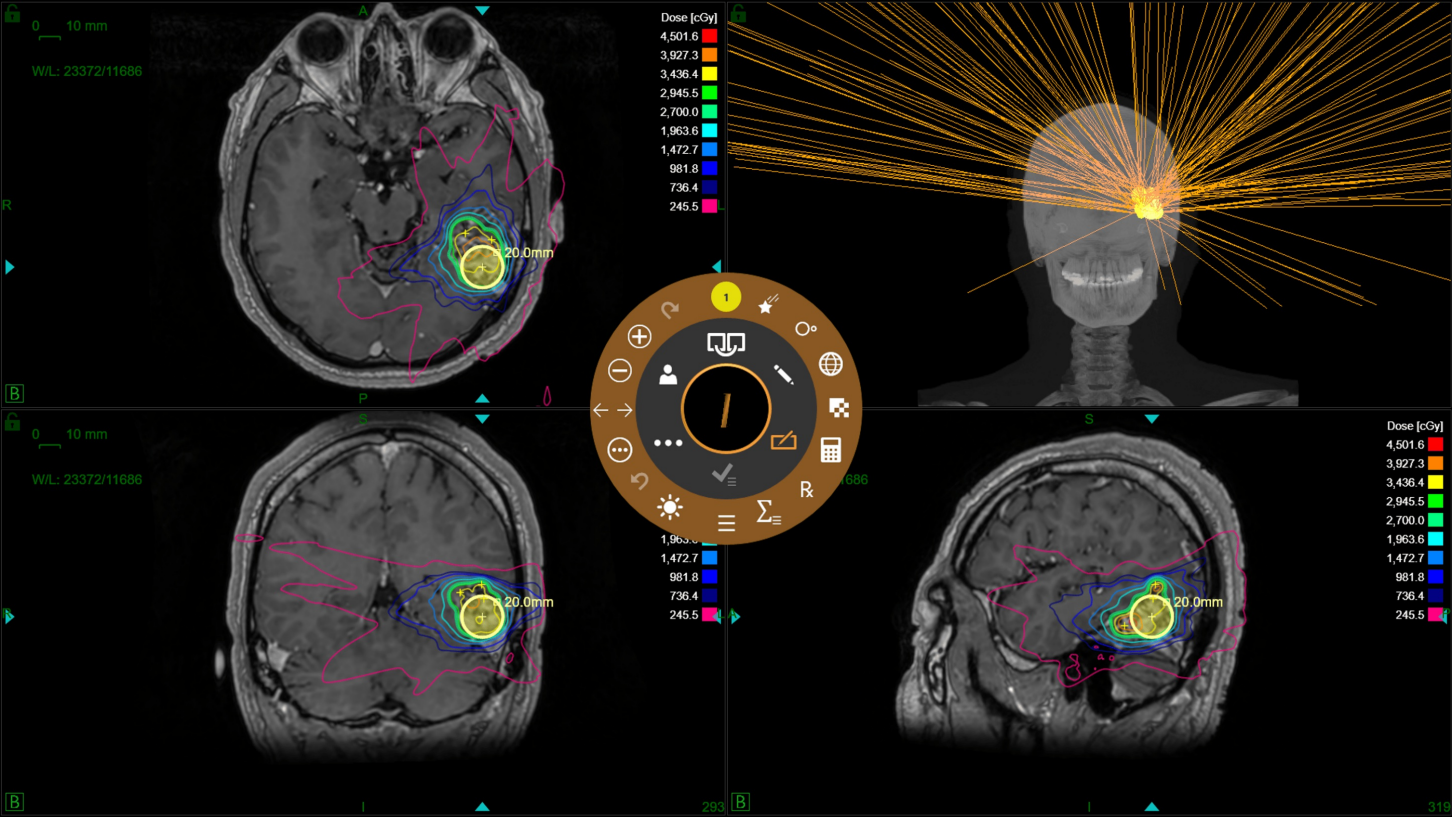
ZAP-X treatment planning software used for patient 1, showing dose delivery visualizations. Image Credit: Michael Chaga

Treatment planning was performed by the departments of neurosurgery and radiation oncology in conjunction with medical physicists. Volumes of interest were contoured using a fused dataset composed of the treatment planning CT and post-gadolinium MRI images within the Eclipse (version 15.6) (Varian Medical Systems, Palo Alto, CA) treatment planning system (TPS). The contoured photos were imported into the ZAP-X TPS (version 1.8 and 1.9). Forward- and inverse-planning was performed, where isocenters were manually placed first, followed by inverse optimization of beam weights and manual addition, deletion, or repositioning of isocenters. A 0.5 mm dose grid was used, limiting the dose to the optic nerves, optic chiasm, cochleae, eyes, brainstem, and spinal cord based on Timmerman organ-at-risk (OAR) recommendations [10]. Plan quality was evaluated for conformity and dose fall-off using the conformity index (CI) and the gradient index (GI). The definition for CI was the ratio of the prescription isodose volume and the target volume [11]. GI was defined as the ratio of half the prescription isodose volume and the prescription isodose volume [12]. Radiation Therapy Oncology Group protocol 0813 was used for plan quality constraints [13]. Patient 1 had a target volume of 10.50 cm^3^ and a plan consisting of eight isocenters, with a prescription dose of 27 Gy in three fractions at the 55% isodose line. The plan used 5, 7.5, 12.5, 15, and 20 mm collimators, path 5 gantry movement, and 177 beams. Patient 2 had a target volume of 4.84 cm^3^ and a plan consisting of 10 isocenters, with a prescription dose of 37.5 Gy in five fractions at the 64% isodose line. The plan used 7.5, 10, 12.5, and 15 mm collimators, path 10 gantry movement, and 289 beams. Patient 3 had a target volume of 11.56 cm^3^ and a plan consisting of 13 isocenters, with a prescription dose of 37.5 Gy in five fractions at the 63% isodose line. The plan used 7.5, 10, 12.5, 15, and 20 mm collimators, path 6 gantry movement, and 318 beams. The ZAP-X treatment plan for patient 1 is demonstrated in Figure 2.

Independent second monitor unit check

Accurate delivery of the prescribed dose is ensured through validation of the dose per monitor unit (MU). To verify these calculations, in-house developed software performs a secondary check to calculate MUs [14]. Originally developed for CyberKnife systems, this software was adapted to incorporate ZAP-X beam data, including cone factors, tissue phantom ratios, and off-center ratios. The tolerance for maximum dose accuracy was ≥95%.

Patient-specific quality assurance

Similarly, patient-specific quality assurance (PSQA) is a critical metric to ensure the efficacy and accuracy of SRS treatments. Before treatment, a PSQA program was implemented to validate the high accuracy of the planned versus delivered dose for each case. PSQA was conducted using SRS MapCHECK^® ^(Sun Nuclear Corporation, Melbourne, FL), simulating treatment plans in the ZAP-X TPS with the following parameters: “Simulate plan to ion chamber target center”; density override of 1.2 g·cm⁻³ for dose calculations; a dose grid of 0.5 mm; and centering the target on the diode detector. Gamma analysis was performed using the SNC Patient (Sun Nuclear Corporation, Melbourne, FL) software, applying 6FFF angular correction, a low-dose threshold of 10%, a distance-to-agreement of 1%, a dose difference of 2%, and gamma passing rate criteria of ≥ 95%.

Machine quality assurance

To verify system functionality, daily quality assurance (QA) tests were performed, beginning with a 6000 MU system warm-up to ensure stability. A Winston-Lutz test was performed to ensure the proper alignment of the radiation beam and mechanical isocenter of the ZAP-X machine. A 4 mm tungsten ball was inserted into a Hayes Phantom with an immobilization mask; this setup was used to test radiation delivery. During the test, beams were delivered isocentrically with a 10 mm collimator at 6 gantry angles. Megavoltage images were captured during the beam delivery to measure the offset between the centers of the radiation field and the shadow of the ball. System geometry equations were used to calculate the ball’s position and the beam’s central axis relative to the ZAP-X mechanical isocenter. The tolerance for these tests was <1 mm. The radiation output was validated using a PTW TN31021 ion chamber placed at the isocenter of the machine and a 25 mm collimator. Radiation output was set at 500 MU and was delivered twice. The tolerance between the expected dose and the measured dose was ±2%. The primary and secondary monitor chamber doses achieved a tolerance difference of ≤3% between the chambers.

Results 

Table 1 summarizes the various dosimetric parameters. Table 2 summarizes plan quality metrics and doses to the target and OARs. All machine QA tests were within the recommended tolerances. Secondary MU check maximum dose accuracy ranged from 99.74% to 99.9%. The gamma passing rate ranged from 97% to 99.6%. The average treatment time per fraction was 38.2±1.6 minutes.

**Table 1:** ZAP-X AVM SRS dosimetric parameters. Average ± standard deviation (range). The setup time includes auto-alignment imaging and review duration. Gantry time is the duration for the gantry to move all gantry angles throughout the entire delivery. Table time is the duration for the treatment table to move to all table locations throughout the entire delivery. kV imaging and processing time is the duration of acquiring the kV images throughout the whole delivery, matching them to DRRs, and performing noise reduction, edge detection, and normalization. Linac time is the beam-on delivery duration. AVM, Arteriovenous malformation; DRR, digitally reconstructed radiograph; MU, monitor unit; SRS, stereotactic radiosurgery

Dosimetric Parameter	Patient 1	Patient 2	Patient 3
Target Volume (cm^3^)	10.50	4.84	11.56
Prescription Dose (Gy)	27	37.5	37.5
Prescription Isodose Line (%)	55	64	63
Fraction Number	3	5	5
Number of Isocenters	8	10	13
Number of Beams	177	289	318
Second MU Check Maximum Dose Accuracy (%)	99.74	99.99	99.75
Gamma Passing Rate (%)	99.6	97	99.1
Delivered MU/Fx	9268.16±0.11 (9268.06–9268.27)	9096.76±0.11 (9096.76–9096.98)	9029.1±0.5 (9028.57–9028.62)
Treatment Time (min/Fx)	55±12 (42.45–67.2)	64±8 (58.37–73.68)	74±6 (73.18–84.13)
Setup Time (min/Fx)	17±12 (6.18–30.3)	11±6 (6.68–18.43)	9±4 (9.88–17.4)
Gantry Time (min/Fx)	27±4 (23.48–30.07)	36±5 (33.01–42)	45±3 (45.72–50.35)
Table Time (min/Fx)	0.69±0.05 (0.63–0.72)	0.75±0.03 (0.73–0.78)	1.11±0.19 (1.03–1.38)
kV Imaging and Processing Time (min/Fx)	3±2 (0.88–5.75)	9±3 (6.05–11.5)	11±2 (8.87–13.33)
Linac Time (min/Fx)	6.41±0.01 (6.4–6.42)	6.44±0.01 (6.42–6.43)	6.41±0.03 (6.42–6.46)

**Table 2:** ZAP-X AVM SRS plan quality metrics and doses to the target and OARs. AVM, arteriovenous malformation; CI, conformity index; D₀.₀₃₅cc, maximum dose to 0.035 cm³; Dmax, maximum point dose; GI, gradient index; GTV, gross tumor volume; OAR, organ at risk; SRS, stereotactic radiosurgery; V100 (%), prescription GTV coverage; V1530cGy, volume receiving 1530 cGy; V1590cGy, volume receiving 1590 cGy; V2200cGy, volume receiving 2200 cGy, V2300cGy, volume receiving 2300 cGy; V2400cGy, volume receiving 2400 cGy; V3100cGy, volume receiving 3100 cGy; V3650cGy, volume receiving 3650 cGy

Dosimetric Parameter	Patient 1 (three fractions)			Patient 2 (five fractions)			Patient 3 (five fractions)		
	Metric	Constraint	ZAP-X AVM SRS	Metric	Constraint	ZAP-X AVM SRS	Metric	Constraint	ZAP-X AVM SRS
GTV	V100 (%)	≥95%	98.33%	V100 (%)	≥95%	97.22%	V100 (%)	≥95%	97.33%
	CI	≤1.2–1.5	1.236	CI	≤1.2–1.5	1.257	CI	≤1.2–1.5	1.176
	GI	≤2.99–3.38	2.857	GI	≤3.6–3.9	3.139	GI	≤2.99–3.38	2.68
Left Optic Nerve	D0.035cc (cGy)	≤1740 cGy	138.5 cGy	D0.035cc (cGy)	≤2500 cGy	104.7 cGy	D0.035cc (cGy)	≤2500 cGy	109.8 cGy
	V1530cGy (cm^3^)	≤0.2 cm^3^	0 cm^3^	V2300cGy (cm^3^)	≤0.2 cm^3^	0 cm^3^	V2300cGy (cm^3^)	≤0.2 cm^3^	0 cm^3^
Right Optic Nerve	D0.035cc (cGy)	≤1740 cGy	85.4 cGy	D0.035cc (cGy)	≤2500 cGy	99.9 cGy	D0.035cc (cGy)	≤2500 cGy	112.6 cGy
	V1530cGy (cm^3^)	≤0.2 cm^3^	0 cm^3^	V2300cGy (cm^3^)	≤0.2 cm^3^	0 cm^3^	V2300cGy (cm^3^)	≤0.2 cm^3^	0 cm^3^
Optic Chiasm	D0.035cc (cGy)	≤1740 cGy	196.7 cGy	D0.035cc (cGy)	≤2500 cGy	256.7 cGy	D0.035cc (cGy)	≤2500 cGy	218.5 cGy
	V2300cGy (cm^3^)	≤0.2 cm^3^	0 cm^3^	V2300cGy (cm^3^)	≤0.2 cm^3^	0 cm^3^	V2300cGy (cm^3^)	≤0.2 cm^3^	0 cm^3^
Left Cochlea	D0.035cc (cGy)	≤1440 cGy	117.6 cGy	D0.035cc (cGy)	≤2200 cGy	249 cGy	D0.035cc (cGy)	≤2200 cGy	99.2 cGy
Right Cochlea	D0.035cc (cGy)	≤1440 cGy	72.5 cGy	D0.035cc (cGy)	≤2200 cGy	117.4 cGy	D0.035cc (cGy)	≤2200 cGy	98.6 cGy
Left Eye	D_max_ (cGy)	≤100–200 cGy	91.4 cGy	D_max_ (cGy)	≤100–200 cGy	86.9 cGy	D_max_ (cGy)	≤100–200 cGy	84.7 cGy
Right Eye	D_max_ (cGy)	≤100–200 cGy	95.6 cGy	D_max_ (cGy)	≤100–200 cGy	87.5 cGy	D_max_ (cGy)	≤100–200 cGy	78.1 cGy
Brainstem (Excluding Medulla)	D0.035cc (cGy)	≤2310 cGy	216 cGy	D0.035cc (cGy)	≤3100 cGy	486.9 cGy	D0.035cc (cGy)	≤3100 cGy	111.7 cGy
	V1590cGy (cm^3^)	≤0.5 cm^3^	0 cm^3^	V2300cGy (cm^3^)	≤0.5 cm^3^	0 cm^3^	V2300cGy (cm^3^)	≤0.5 cm^3^	0 cm^3^
Spinal Cord (Including Medulla)	D0.035cc (cGy)	≤2250 cGy	195.2 cGy	D0.035cc (cGy)	≤2800 cGy	159.6 cGy	D0.035cc (cGy)	≤2800 cGy	99 cGy
	V1590cGy (cm^3^)	≤0.35 cm^3^	0 cm^3^	V2200cGy (cm^3^)	≤0.35 cm^3^	0 cm^3^	V2200cGy (cm^3^)	≤0.35 cm^3^	0 cm^3^
Skin	D0.035cc (cGy)	≤3300 cGy	1093 cGy	D0.035cc (cGy)	≤3850 cGy	1513 cGy	D0.035cc (cGy)	≤3850 cGy	877.5 cGy
	V3100cGy (cm^3^)	≤10 cm^3^	0 cm^3^	V3650cGy (cm^3^)	≤10 cm^3^	0 cm^3^	V3650cGy (cm^3^)	≤10 cm^3^	0 cm^3^
Brain–GTV	V2400cGy (cm^3^)	≤16.8 cm^3^	5.339 cm^3^	V2400cGy (cm^3^)	≤16.8 cm^3^	7.414 cm^3^	V2400cGy (cm^3^)	≤16.8 cm^3^	12.68 cm^3^

**Figure 3:**
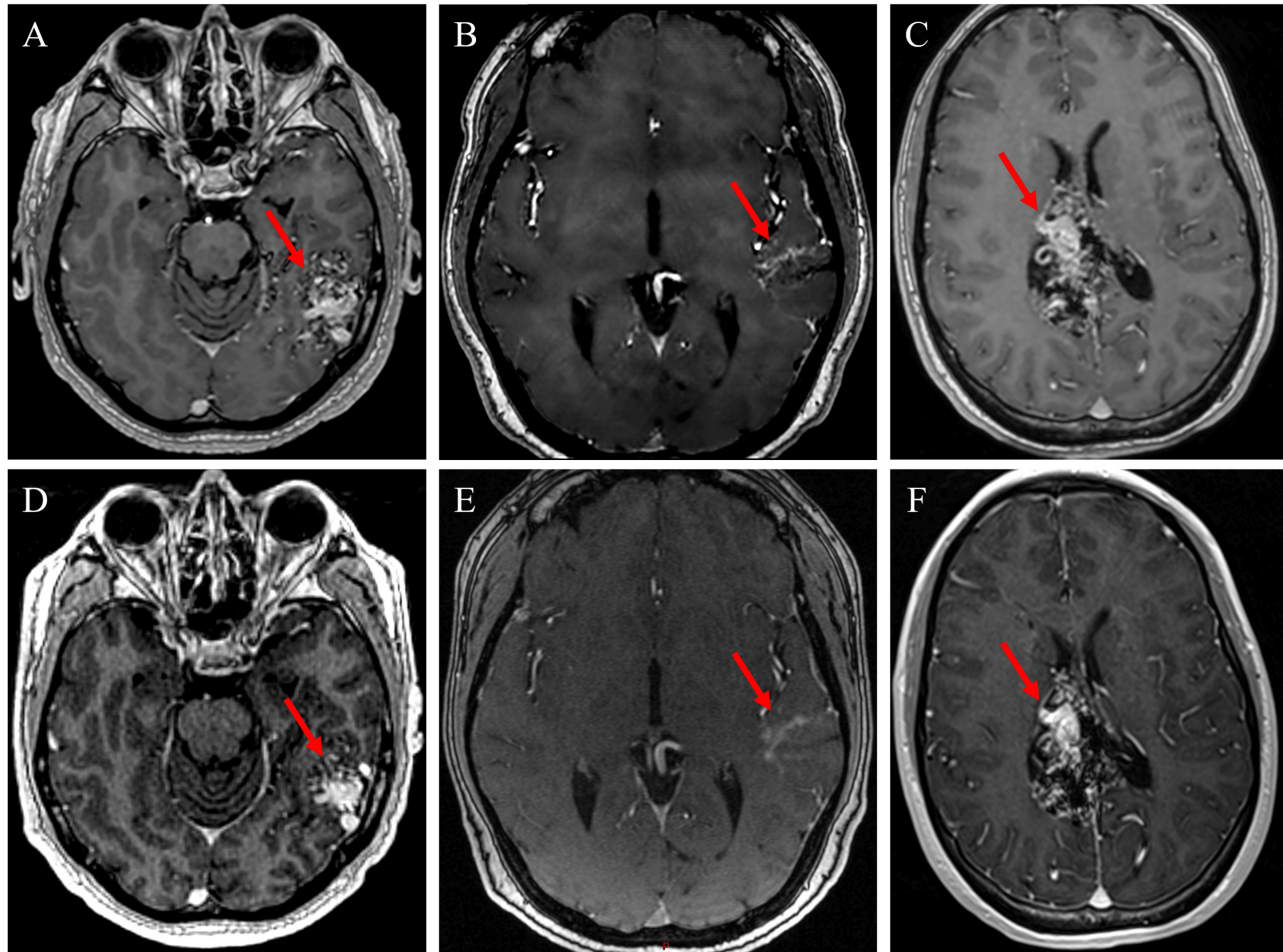
Comparison of pre- and post-treatment MRIs: A) Patient 1: pre-treatment T1 MRI, B) patient 2: pre-treatment MRA, C) patient 3: pre-treatment T1 MRI, D) patient 1: one-year post-treatment T1 MRI, E) patient 2: four-month post-treatment MRA, F) patient 3: six-month post-treatment T1 MRI. Image Credit: Michael Chaga MRA, magnetic resonance angiography

Patient 1 tolerated the treatment well with no side effects. At a follow-up visit two months postoperatively, he reported experiencing one episode of headache and visual disturbance; these symptoms resolved with steroid treatment in three days. Upon one-year follow-up postoperative imaging, radiology reported AVM size improvement on both MRI and MRA, measuring 3.0×2.5 cm (Figures 3A, 3D). The patient reported that he was doing well, with no headaches, visual changes, dizziness, or other neurological symptoms. 

Patient 2 tolerated treatment well, with visual symptoms resolved and no side effects. At her one-month follow-up visit, she reported that she was doing very well since treatment and was able to carry out her daily routines. She denied any new complaints. The patient presented to the ED for a new-onset seizure; epilepsy was determined, and her epileptiform activity was likely related to the AVM, and she was started on Keppra. Upon four-month follow-up postoperative imaging, radiology reported AVM size improvement on both MRI and MRA, measuring 2.7×1.5 cm (Figures 3B, 3E). The patient reported that she was doing well, with no more visual defects or other neurological symptoms.

Patient 3 tolerated the treatment well, with no episodes of visual changes, headaches, or other SRS-related side effects. At her six-week follow-up appointment, she was found to be well and denied any new neurological symptoms. Upon six-month follow-up postoperative imaging, radiology reported stable MRI and MRA AVM size, measuring 5.7×2.9 cm. The patient reported that she was doing well, with no new neurological symptoms (Figures 3C, 3F).

## Discussion

Current treatment options for AVMs include observation, embolization, radiosurgery, and microsurgery. AVMs are assessed and classified according to the Spetzler-Martin grading, with higher grade malformations requiring different management strategies. Embolization is often indicated for higher-grade AVMs and is often used in conjunction with SRS, microsurgery, or both. SRS has been shown to be successful in the obliteration of high-grade AVMs, both with and without prior embolization [5]. Of our three patients, one had undergone multiple embolizations before the addition of SRS to minimize the AVM volume. While these embolizations did not result in a reduction in size, treatment with the ZAP-X platform was confirmed to have shrunk the AVM volume in this particular case. Reducing AVM size is critical to reduce the risk of rupture and to prevent mass effect upon adjacent structures. As such, this finding preliminarily demonstrates the utility of ZAP-X in lowering the risk of such symptoms. 

AVMs that are more vascular have a higher risk of bleeding and rupture due to increased stress on vessel walls [15,16]. Thus, malformations that have greater vascular outlet require more aggressive therapy to ensure such events do not ensue. SRS, particularly Gamma Knife, has been proven to reduce long-term hemorrhage risk compared to conservative management [17]. Thus, management of bleeding risk for AVMs with SRS is an important consideration for such patients. Our results show the feasibility and applicability of using the ZAP-X system to reduce such risk. 

The specificity of SRS plays a pivotal role in the management of AVMs due to the ability to balance therapeutic efficacy with the preservation of normal brain function. Precise targeting of the AVM nidus minimizes the dose delivered to surrounding brain tissue, significantly reducing the risk of long-term complications, including radiation-induced neurological deficits. The literature indicates that a dose greater than 12 Gy to adjacent normal brain structures is associated with a 5.5% seven-year actuarial rate of developing persistent symptomatic neurological deficits [18]. Our study demonstrated the effectiveness of advanced imaging, dose shaping, and careful targeting in mitigating this risk, as none of our patients reported complications or side effects related to toxicity. This outcome underscores the importance of precise treatment planning in achieving safe and effective outcomes for patients with AVMs, particularly when they are located near eloquent brain areas.

ZAP-X exemplifies how advancements in SRS technology can optimize treatment specificity for AVMs. The ZAP-X system’s capabilities, including real-time imaging and multiangle beam delivery, enhance the precision of dose delivery to the AVM while limiting radiation exposure to surrounding areas. This technology is particularly beneficial for treating AVMs in complex anatomical locations, as demonstrated in our cases, where the patients tolerated treatment without significant side effects and exhibited improved or stable clinical outcomes. For example, patients 1 and 2 experienced resolution of pre-treatment symptoms, such as headaches and visual disturbances, following ZAP-X SRS. This symptomatic improvement, combined with the absence of treatment-related complications, highlights the broader potential of SRS to not only manage the structural aspects of AVMs but also address associated clinical symptoms.

SRS treatment strategies for large AVMs (>10 cm^3^) involve the delivery of radiation doses in stages with dose- or volume-staged SRS. Dose staging is described in the literature as either hypofractionated or repeat SRS. In volume-staged SRS, distinct geometrical portions of the AVM are treated over time until the entire AVM is irradiated. Both dose- and volume-staged SRS are used to facilitate obliteration while reducing complication rates for large AVMs [19]. In this study, patients 1 and 3 have large AVMs that were treated with dose-staged SRS. In a systematic literature review, Moosa et al. reported mean rates for dose- and volume-staged groups of 22.8% and 47.5% for complete obliteration, 13.5% and 13.6% for symptomatic radiation-induced changes, 12.3% and 17.8% for cumulative post-SRS latency period hemorrhage, and 3.2% and 4.6% for post-SRS mortality [19]. In a systematic literature review, Larkin et al. reported mean rates for dose- and volume-staged groups of 46.6% and 17.8% for complete obliteration and 18% and 23.6% for complication rates [20].

The goal of AVM SRS is typically complete obliteration of the AVM nidus to prevent further intracranial hemorrhage and neurological deficits. Complete obliteration typically occurs 3-5 years after treatment for AVM SRS [21]. In a systematic review and meta-analysis on hypofractionated AVM SRS, Jaikumar et al. reported a 50.1% obliteration rate at 41.2 months of follow-up, with an associated 11.1% rupture rate, 5.5% new-onset seizure rate, 10.4% radionecrosis, and 6% AVM-related mortality. Comparing doses of ≥35 Gy with those of <35 Gy demonstrated trends toward higher rates of obliteration and radionecrosis with higher doses [22]. In a study on Linac-based AVM SRS, Gawish et al. reported that after a follow-up of one, two, and three years, the obliteration rates were 22%, 59%, and 66% [23].

## Conclusions

The initial experience using the ZAP-X gyroscopic SRS system for treating brain AVMs demonstrates promising clinical and radiographic outcomes. Across all three patients, the treatment was well tolerated, with no significant acute side effects, and favorable early imaging results showed either reduction or stability in AVM size. Importantly, the individualized treatment plan allowed for successful dosing tailored to the AVM location and patient anatomy, highlighting the precision and adaptability of the ZAP-X platform. This system is an effective tool for managing AVMs, particularly in patients with complex or surgically inaccessible lesions. The precision of ZAP-X, enabled by its gyroscopic beam delivery, real-time imaging, and self-shielding design, represents a significant advancement in the non-invasive management of AVMs. These features contribute to accurate target coverage while minimizing the radiation dose to surrounding critical structures, lowering the potential long-term neurologic complications. As elucidated by our study cohort, even patients with high-grade or previously embolized AVMs benefited from the system's ability for high conformity and dose gradient indices, reinforcing the utility of the ZAP-X system for both primary and adjunctive therapy in multimodal AVM management.

Although our study adds valuable insight into the clinical application of this next-generation neurological system, continued follow-up and larger prospective studies are needed to validate long-term obliteration rates and neurological outcomes. Our findings support the feasibility, safety, and therapeutic potential of ZAP-X SRS in the treatment of cerebral AVMs and highlight the importance of expanding access to this innovative and highly precise SRS option.
